# Synthetic Pesticides Used in Agricultural Production Promote Genetic Instability and Metabolic Variability in *Candida* spp.

**DOI:** 10.3390/genes11080848

**Published:** 2020-07-24

**Authors:** Leszek Potocki, Aleksandra Baran, Bernadetta Oklejewicz, Ewa Szpyrka, Magdalena Podbielska, Viera Schwarzbacherová

**Affiliations:** 1 Department of Biotechnology, College of Natural Sciences, University of Rzeszow, Pigonia 1, 35-310 Rzeszow, Poland; lenno1998@wp.pl (A.B.); b.oklejewicz@gmail.com (B.O.); ewaszpyrka@interia.pl (E.S.); magdapodbiel@gmail.com (M.P.); 2 Department of Biology and Genetics, Institute of Genetics, University of Veterinary Medicine and Pharmacy in Košice, Komenského 73, 041 81 Košice, Slovak

**Keywords:** *Candida*, pesticide, metabolic activity, genetic damage, cell growth

## Abstract

The effects of triazole fungicide Tango^®^ (epoxiconazole) and two neonicotinoid insecticide formulations Mospilan^®^ (acetamiprid) and Calypso^®^ (thiacloprid) were investigated in *Candida albicans* and three non-albicans species *Candida pulcherrima, Candida glabrata* and *Candida tropicalis* to assess the range of morphological, metabolic and genetic changes after their exposure to pesticides. Moreover, the bioavailability of pesticides, which gives us information about their metabolization was assessed using gas chromatography-mass spectrophotometry (GC-MS). The tested pesticides caused differences between the cells of the same species in the studied populations in response to ROS accumulation, the level of DNA damage, changes in fatty acids (FAs) and phospholipid profiles, change in the percentage of unsaturated to saturated FAs or the ability to biofilm. In addition, for the first time, the effect of tested neonicotinoid insecticides on the change of metabolic profile of colony cells during aging was demonstrated. Our data suggest that widely used pesticides, including insecticides, may increase cellular diversity in the *Candida* species population-known as clonal heterogeneity-and thus play an important role in acquiring resistance to antifungal agents.

## 1. Introduction

Pesticides comprise a wide range of chemicals of different classes used to ensure higher crop yields, but on the other hand, many adverse effects have been detected in animals and humans after acute or chronic exposure to pesticides, including cancer [1,2], lower fertility [3,4], metabolic changes [5,6] and changes in gastrointestinal microbiomes [7,8]. Symbiotic microorganisms play crucial roles in many important processes, such as vitamin synthesis [9], energy metabolism [10], neurodevelopment [11] and immune system modulation [12]. If there are disbalances and the immune system is weakened, facultative pathogens may cause disease. A typical representative is the genus *Candida*, which is normally present on the mucous membranes and keeps in balance with other host microbiota. After injury, candidiasis is commonly detected in the oral cavity, urogenital tract, on the skin, or as a systemic fungal disease in humans and animals [13,14]. *Candida* yeast is a heterogeneous group of microorganisms, inhabiting various environments. The genus *Candida*, belonging in the family *Debaryomycetaceae* within the subtype *Saccharomycotina*, type *Ascomycota*, includes over 200 morphologically diverse species; their common feature is the ability to reproduce asexually through budding [15,16]. Apart from pathogenic *Candida* spp. there are beneficial species, which are often involved in the fermentation processes of sausage, bread, coffee beans and cheese production [17], or they are detected on the surfaces of fruit and vegetables [18]. In the soil, they play an important role in nutrient transformation, plant growth promotion, and plant protection against some diseases [19].

On the other hand, it is well known that the microbiota is very sensitive to diet, environmental pollutants and drug administration. Xenobiotics may be metabolized by the gastrointestinal microbiota and metabolites can lead to increased [20] or reduced toxicity [21].

Mechanisms of antifungal activity of fungicides as well as pesticides mechanism of action on pests are mostly understood [22,23,24,25].

On the other hand, soil fungi are often exposed to chemicals used in agriculture and among them not only to fungicides but also to other pesticides. There is a current gap in knowledge on how insecticides, not only fungicides, may modulate fungal cells’ heterogeneity. In addition, there is still a lack of information about the impact of pesticides on microbiological flora after entering the human and animal body.

This study aimed to assess the impact of commonly used pesticides as an underestimated and neglected source of cellular variability of microorganisms, including yeast of the *Candida* genus, which may lead to the creation of clonal heterogeneity with altered morphological, genetic and physiological profiles. The consequence of this mechanism may be the creation of a population of cells with new features (e.g., acquisition of drug resistance) in relation to the initial population. Furthermore, to make our conclusions more general we chose also *Candida* strains isolated from infected humans, *C. pulcherrima* isolated from the environment and *C. albicans ATCC 14053*, the most defined strain, as a control.

In our experiments, a fungicide and two insecticide formulations were tested. The twin-component fungicide formulation Tango^®^ Star (84 g·L^−1^ epoxiconazole and 250 g·L^−1^ fenpropimorph) was applied to cereal crops and sugar beet to prevent fungal diseases. Its active agent, epoxiconazole, belonging in the group of triazoles, acts as an inhibitor of lanosterol 14-α-demethylase (CYP 51). This enzyme is necessary for ergosterol synthesis, the basic steroid component of the fungal cell membrane [26]. The second active agent, a morpholine fenpropimorph, inhibits ergosterol synthesis through *ERG2* and *ERG24* gene inhibition. The product of the *ERG2* gene catalyzes the change of zymosterol to episterol and the product of the *ERG24* gene catalyzes the change of dimethyl-cholestratrienol to fecosterol in the ergosterol biosynthesis process [27]. Additionally, different levels of yeasts and the composition of their communities were detected after fungicide treatment. The main reason for these differences is the varying susceptibility of the yeasts to the applied fungicides [28]. Next, neonicotinoid insecticides Calypso^®^ 480SC (480 g·L^−1^ thiacloprid) and Mospilan^®^ 20SP (20% acetamiprid) were examined. Both are widely used to protect fruits, vegetables and ornamental plants against various insects. Their active agents act as selective agonists to nicotinic acetylcholine receptors in insects, while lower toxicity is observed in the vertebrates including humans due to the lower occurrence of these receptors [29,30].

## 2. Materials and Methods

### 2.1. Candida Strains, Experimental Conditions and Pesticides Tested

The *Candida* strains used in this work are listed in Table 1. For all experiments, all *Candida* strain pre-cultures for biomass propagation were grown on liquid YPD medium (1% *w/v* Difco Yeast Extract, 2% *w/v* Difco Yeast Bacto-Peptone, 2% *w/v* dextrose). Liquid cultures were incubated at 28 °C for 24 h using an orbital shaker (120 rpm).

Three commercially available pesticide formulations were bought in Poland and used in our experiment, namely the fungicide Tango^®^ Star (250 g·L^−1^ fenpropimorph and 84 g·L^−1^ epoxiconazole) and two insecticides, Mospilan^®^ 20 SP with active ingredient acetamiprid (≥ 2 g·L^−1^) and Calypso^®^ 480 SC with active ingredient thiacloprid (480 g·L^−1^). All pesticides were dissolved in sterile water and were added to cell cultures at final concentrations of 6, 12 and 25 µg·mL^−1^ for Tango^®^; 25, 50 and 100 µg·mL^−1^ for Mospilan^®^ and 60, 120 and 250 µg·mL^−1^ for Calypso^®^. Final concentrations for individual compounds were determined based on kinetic growth analysis. In some experiments (cell viability and oxidative stress detection), the effect of pure active agents alone or in combination was also analyzed. The following concentrations were used for epoxiconazole: 1.51, 3.02 and 6.29 µg·mL^−1^; fenpropimorph: 4.49, 8.98 and 18.71 µg·mL^−1^ or their mixture; acetamiprid: 25, 50 and 100 µg·mL^−1^; and thiacloprid: 60, 120 and 250 µg·mL^−1^.

### 2.2. Testing Sensitivity to Pesticide Treatment

The effects of the pesticides on yeasts were tested in liquid culture. For this purpose, the yeast cells were washed, and suspended in YPD medium (total volume 250 μL) with a working concentration of 5 × 10^6^ cells·mL^−1^ and cultured in a 96-well format shaker (900 rpm) at 28 °C with the addition of the specified concentrations of pesticides. Five different concentrations of every substance were examined; for Tango^®^ and Mospilan^®^ 6; 12; 25; 50 and 100 µg·mL^−1^ and for Calypso^®^ 60; 120; 250; 500 and 1000 µg·mL^−1^.The final concentrations tested for individual compounds were determined based on kinetic growth analysis.

### 2.3. Growth Rate

For the kinetics of the growth assay, *Candida* cells were washed and suspended in a YPD medium to a total volume of 250 μL with a working concentration of 5 × 10^6^ cells·mL^−1^ and cultured in a 96-well format shaker (900 rpm) at 28 °C with the addition of the final concentrations tested. The optical density (OD) at 600 nm was determined for each well using a Tecan Scientific microplate reader equipped with monochromator optics every hour during an 8h period.

### 2.4. Morphology Assessment

To assess the morphological characteristics of yeast strains, the cells of tested isolates cultured with specific concentrations of pesticides were centrifuged and then diluted with 0.9% NaCl (Sigma-Aldrich, Poznan, Poland). The effects of the pesticides on the morphology of individual *Candida* species were determined using an Olympus light microscope equipped with a DP72 CCD camera and Olympus CellF software. Observations were made under the magnification of a 100 × lens.

### 2.5. Effect on Candida Colony Aging

Cells were plated on GM–PKB agar (1% yeast extract, 3% glycerol, 2% agar, 30 mM CaCl_2_, 0.01% BKP) with the addition of selected concentrations for Tango^®^ (6 µg·mL^−1^), Mospilan^®^(100 µg·mL^−1^) and Calypso^®^-(250 µg·mL^−1^) at densities of 10^3^ per plate; the lowest inhibitory concentration of each pesticide tested was chosen. The plates with cells were incubated at 28°C for 50 days. Color changes in GM–PKB agar were observed after 3, 7, 12, 15 and 50 days of incubation and recorded using a Nikon D850 Digital SLR Camera.

### 2.6. Biofilm Assay

Cells (1 × 10^7^) were taken and suspended in a YPD to a final volume of 1 mL. Then 250 µL of culture was applied to a 96-well plate and incubated at 37 °C for 48 h. After incubation, the culture was gently removed with a multichannel pipette and rinsed with sterile 1 × PBS. The biofilm was stained with 0.4% crystal violet for 15 min. The dye was then removed, the excess was washed off with distilled water and the plate was dried at room temperature (RT). Next, 200 µL of 95% ethanol was added to each well in the plate and absorbance was measured using a Tecan Scientific microplate reader equipped with monochromator optics at 595 nm wavelength.

### 2.7. Pesticide Content Analysis in Medium and Cell Pellet

After pesticide treatment, cell pellet and medium were analyzed using the gas chromatography-mass spectroscopy (GC-MS) method to detect where the pesticides predominantly occurred, meaning whether they were metabolized by the cells or not. Initially, 1 mL of supernatant was placed in a 15 mL propylene centrifuge tube and 5 mL acetone was added. Then 0.2 g Na_2_SO_4_ was added and the contents of the centrifuge tube were mixed for 1 min (BenchMixerTM, Benchmark- Scientific, Inc., Edison, NJ, USA). Next, 0.2 mL of extract was put into 2 mL vials and 0.8 mL of petroleum ether and 0.1 mL of internal standard (for GC-MS) were added. To the pellet, 0.5 mL of acetone was added (in 2 mL tubes) and mixed for 8 min. After centrifugation (3000 rpm, 5 min) the whole extract was put into 2 mL vials and 0.1 mL of internal standard was added (for GC-MS) [31].

### 2.8. Cell Viability Assays

Cell viability was estimated with a LIVE/DEAD^®^ Yeast Viability Kit (Molecular Probes, Leiden, Netherlands) using the standard protocol according to the manufacturer’s instructions. Briefly, cells of each strain were washed and stained with a mixture of FUN^®^1 and Calcofluor^®^ White M2R and inspected under an Olympus BX61 fluorescence microscope equipped with a DP72 CCD camera and Olympus CellF software. A total of 200 cells were used for the analysis.

The antimicrobial properties of pesticides in liquid yeast cultures were determined using the Alamar Blue (resazurin) cell viability assay according to Schneemann et al. [32]. The principle of this method is based on the irreversible reduction of blue dye resazurin to red-fluorescent resorufin only by metabolically active cells. Briefly, overnight cultures of tested microorganisms in YPD medium (yeast extract—10 g·L^−1^, peptone—20 g·L^−1^, glucose—20 g·L^−1^, pH 7.2) were diluted, counted and suspended in YPD medium (total volume 250 μL) with a working concentration of 5 × 10^6^ cells·mL^−1^ and cultured in a 96-well format shaker (900 rpm) at 28 °C with the addition of the specified concentrations of pesticides. In addition, a negative control (no compound) and a positive control (cycloheximide, 1 mg·mL^−1^) for *Candida* species strains were prepared. After 24 h of incubation, 10 µL of resazurin solution (0.2 mg·mL^−1^ in PBS) was added to each well of the 96-well plate and incubated for 2 h (until the negative control color changed from blue to red/pink). To assess the impact of the analyzed pesticides on the metabolic activity of yeast, a fluorescence measurement at 560 nm after excitation at 590 nm was taken.

### 2.9. Cell Cycle Phase Determination

Fluorescence-activated cell sorting (FACS) analyses were done using an Amnis^®^FlowSight^®^ flow cytometer and IDEAS software version 6.2.187.0 (Merck Millipore, Warsaw, Poland). Briefly, fixed cells in 70% ethanol were incubated with RNase A (1:1 in TE buffer, 1 h, 37 °C) and then digested with proteinase K (1: 1) with Syber Green (1 µL stock solution in DMSO per 1 mL buffer) overnight at 4 °C. After incubation, stained cells were washed 1 × in TE (1:1), then the resulting pellet was suspended in 100 to 200 µL TE (1:1). For each FACS assay, the DNA content of 10,000 single cells was monitored.

### 2.10. Oxidative Stress and Genotoxic Damage Assessment

After 24 h *Candida* cell treatment with commercial pesticides and their active ingredients, the mitochondrial superoxide level (MitoTracker^®^ Red CM H2XRos, Thermo Fisher Scientific, Waltham, MA, USA.) was assessed as previously described [33].The integrated fluorescence density was measured using a Tecan Scientific microplate reader equipped with monochromator optics at excitation wavelength 579 nm and emission 599 nm.

Alkaline comet assay was conducted to assess the potential genotoxic damage in cells [34]. After 24-h treatment with pesticides, cells were processed as previously described by Lewinska et al. [35]. Subsequently, slides were stained with 0.25 µM YOYO-1 (Invitrogen Corporation, Grand Island, NY, USA) in 2.5% DMSO and 0.5% sucrose and mounted with a coverslip and digital comet images were immediately captured with an Olympus BX61 fluorescence microscope equipped with a DP72 CCD camera and Olympus CellF software; YOYO-1 fluorescent signals were collected using FITC filter (λex = 491 nm, λem = 509 nm). At least 100 comets were measured per each sample triplicate using CometScore Software downloaded from http://rexhoover.com/index.php?id=cometscore. The Olive Tail Moment (OTM) was scored as a general parameter for DNA integrity assessment [36] which is the distance (in direction) between the center of gravity of the head (CGH) and the center of gravity of the tail (CGT) and percent tail DNA (DNAT):

OTM = (CGT − CGH) × % DNAT

### 2.11. Total Lipids Content Analysis

Total lipids content was analyzed based on fatty methyl esters according to Wychenand Laurens [37]. Sample preparation started with inserting 20 mg of yeast (dry weight) into a 2 mL chromatographic vial. Then, 25 μL of internal standard C15:0 (1000 μg·mL^−1^), 200 μL of dichloromethane/methanol (2:1, *v/v*) and 300 μL of 0.6 M HCl in methanol were added to the sample. The vials were sealed (PTFE caps), the contents of the vial were shaken by hand and placed in a laboratory oven (SLW 53 SIMPLE, POL-EKO-APARATURA SP.J., Wodzislaw Sl., Poland) andheated to 85 °C ± 3 °C for 1 h. After this time, the vials were cooled (15 min) to RT. Then the isolation of fatty acid esters and preparation of samples for chromatographic analysis were performed. After cooling, 1 mL of petroleum ether was added to the vial, the contents of the vial were shaken by vortex (BenchMixerTM, Benchmark, Scientific, Inc., Edison, NJ, USA) for 5 min and allowed to separate for 1 h. Next, 100 μL of the upper phase was transferred to a 2 mL chromatographic vial and 400 μL of petroleum ether was added. Chromatographic analysis was carried out using a gas chromatograph with a mass detector in full scan mode. Ions from 50 m/z (mass to charge ratio) to 400 m/z were monitored, source temperature 230°C, electron ionization type (EI), temperature program 40–260 °C, column HP-5 MS (Ultra Inert/30 m × 0.25 mm I.D. × 0.25-μm). The linearity was determined based on six-point calibration curves (R^2^ from 0.925 to 0.999). The analysis results include recovery of the method based on the recovery of the added internal standard.

### 2.12. Phospholipid Determination by Phosphorus Assay

Phospholipids from *Candida* cells were extracted hot with an ethanol/ether (3:1) mixture. After evaporation of the solvent, the extract was mineralized, as a result of which phospholipid phosphorus was transformed into orthophosphate. In an acid medium, orthophosphate forms are created with molybdate (VI) and ammonium phosphomolybdate (VI). This compound is reduced to mixed molybdenum oxides, so-called molybdenum blue (MO_2_ × MO_3_), the quantity of which was determined by means of colorimetry at 720 nm. The phosphorus determination in 1 mL (0.01 mg P) of the standard solution was performed in an identical manner.

### 2.13. Glycogen Accumulation Analysis

Analysis of glycogen accumulation was performed according to Chester [38] based on the reddish-brown color of yeasts stained with iodine. Briefly, cells (10^3^ densities) were plated on YPD agar (yeast extract—10 g·L^−1^, peptone—20 g·L^−1^, glucose—20 g·L^−1^, 1.8% to 2% bacteriological agar, pH 7.2) previously incubated for 24 h with the appropriate concentrations tested. After 48 h of incubation at 30 °C, colonies were stained using 5 mL iodine solution (0.2% I_2_ in 0.4% KI). Staining reactions were recorded 1 min after adding the iodine solution. Pictures were taken with a NIKON D850 Digital SLR Camera.

### 2.14. Statistical Analysis

The results are presented as mean ± SE. A simple analysis of variance (ANOVA) and Dunnett’s a posteriori test were used to analyze differences between control and pesticide-treated samples.

## 3. Results

### 3.1. Growth Change, Morphology and Aging

The influence of fungicides to limit the growth of yeasts is well known [39]; however, nothing is known about the influence of insecticides on this process. To this aim, we evaluated the impact of the tested pesticides on the growth kinetics of the analyzed *Candida* spp. during the 8-h period.

**Figure 1:**
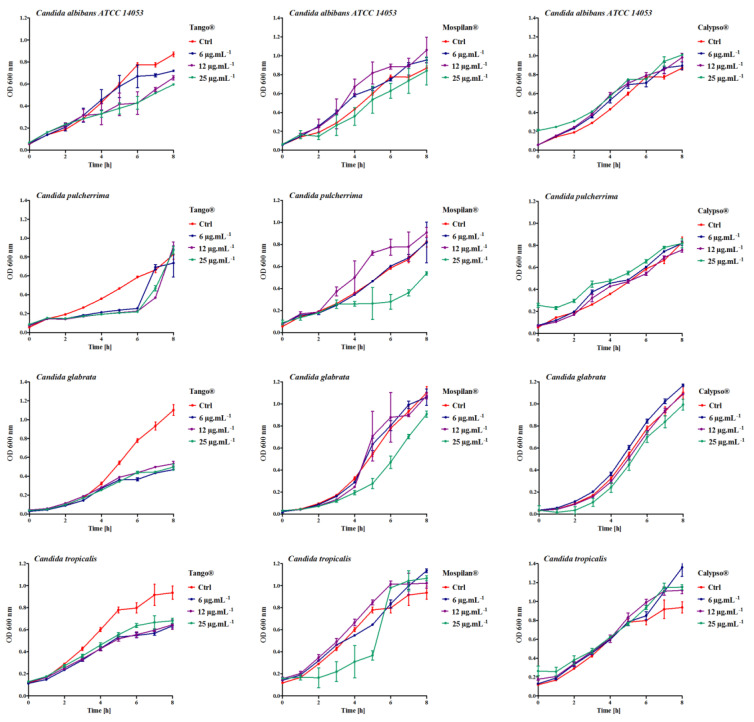
Kinetics of growth of *C. albicans ATCC14053*, *C. pulcherrima*, *C. glabrata*, *C. tropicalis* after treatmentwith Tango^®^, Mospilan^®^, and Calypso^®^. Growth of *Candida* spp. after pesticide treatment was monitored turbidimetrically at 600 nm in a microplate reader every 2 h during an 8h period. Bars indicate SD, *n* = 6.

A comparative analysis of the growth kinetics of the tested *Candida* strains showed their varied sensitivity when exposed to different concentrations and types of pesticide (here we show differences in growth after an 8h incubation with pesticides—Figure 1). No death phase was observed even at the highest pesticide concentrations. However, Tango^®^ fungicide caused a decrease in the number of cells in all tested strains starting from three hours of culture and Mospilan^®^ at a concentration of 100 µg·mL^−1^. Interestingly, despite the delayed growth profile in *Candida pulcherrima* in the initial stages of incubation with Tango^®^ fungicide, after 6 h of treatment growth stimulation was observed compared to the control. After 8 h of growth, *Candida pulcherrima* exhibited the highest tolerance in the highest fungicide concentrations used (12 and 25 µg·mL^−1^). All tested strains adapted to growth in the presence of Calypso^®^ pesticide.

**Figure 2:**
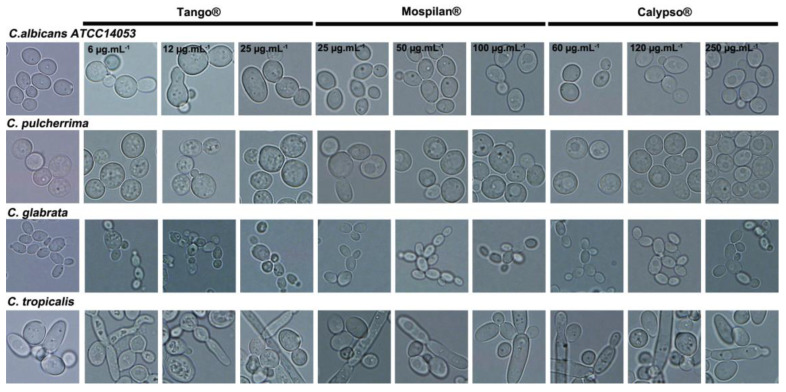
Cell morphology of *C. albicans ATCC 14053*, *C. pulcherrima*, *C. glabrata*, *C. tropicalis* after Tango^®^, Mospilan^®^, and Calypso^®^ treatment. Representative microphotographs are shown; objective 100×.

It was previously shown that not only fungicide Tango^®^ but also insecticides may have inhibited the growth of *Candida* spp. For this reason, in the next stage of research, we decided to check morphological changes in tested strains under the influence of applied insecticides compared to controls (Figure 2). In controls, all *Candida* strains had a typical spherical to oval shape. No changes were detected in *C. pulcherrima* and *C. glabrata* after any pesticide exposure. They tended to create blastospores, with *C. glabrata* producing the smallest. Microscopic observation of the cell morphology of *C. albicans* and *C. pulcherrima* showed an increase in cell size after using each of the Tango^®^ fungicide concentrations. Additionally, *C. albicans* was not able to create hyphae. The twin-component fungicide Tango^®^ which inhibits ergosterol synthesis may contribute to the exhaustion of the intracellular pool of ergosterol while blocking the transition of blastospores to hypha form. On the other hand, pesticide treatment promoted the formation of *C. tropicalis* hyphae and pseudohyphae, mostly seen after Tango^®^, Mospilan^®^ and Calypso^®^ exposure (in each concentration used).

**Figure 3:**
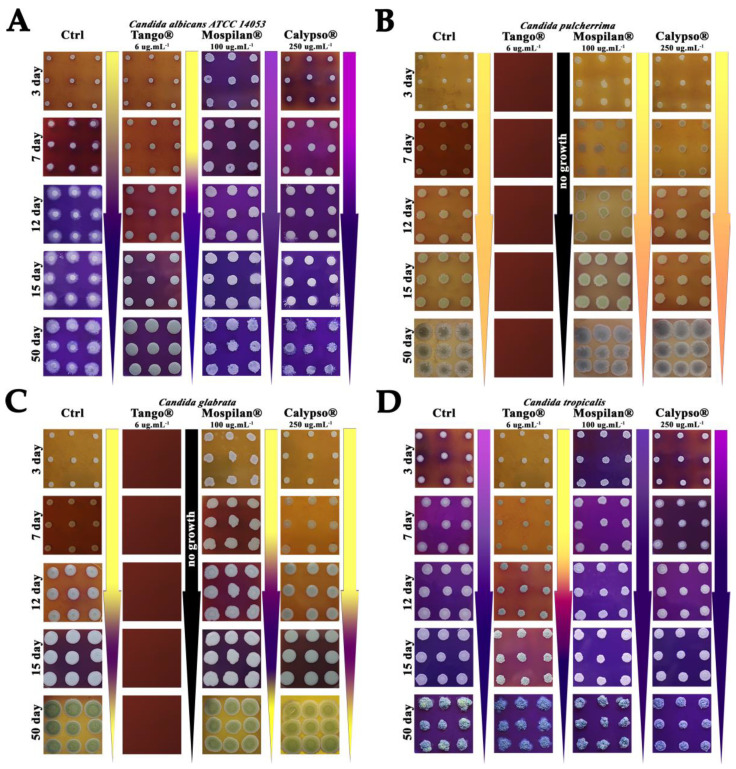
Impact of tested pesticides on the development of colonies formed by (**A**) *C. albicans ATCC 14053,* (**B**) *C. pulcherrima,* (**C**) *C. glabrata,* and (**D**) *C. tropicalis.* Colonies developed on complex glycerol agar with 6 µg·mL^−1^ Tango^®^, 100 µg·mL^−1^ Mospilan^®^, 250 µg·mL^−1^ Calypso^®^. Bromocresol purple, pH dye indicator with pKa 6.3 was used, with the color changing from yellow at acidic pH to purple at a more alkaline pH.

Yeast colonies are a promising model for studying various aspects of microbial multicellularity [40]. Therefore, in the next stage, we decided to assess the influence of the tested pesticides on the developmental phases of the colonies of the tested *Candida* spp. during aging, where cells with high density undergoing metabolic changes may imitate the state of infection in vivo or yeast colonies occurring in the natural environment. During the tests, selected combinations of pesticide concentrations for the growth of individual *Candida* species on a solid medium with glycerol and bromocresol purple (GM-BKP agar – (GM-agar (1% yeast extract, 3% glycerol, 2% agar, 30 mM CaCl_2_), 0.01% bromocresol purple) were tested. The analysis showed varied effects of the pesticides used in communication between the colonies by changing the development patterns of the colonies observed, relative to control. The use of Tango^®^ fungicide at a concentration of 6 μg·mL^−1^ extended the active colony-growing phase (acid phase) to 12 days, after which the alkaline phase persisted until the 50th day of culture (Figure 3A). Under control conditions, colony production of ammonia began from day seven, intensively changing the pH of the medium after 14 days. In addition, a significant increase in colony biomass with a smooth phenotype was observed on the fungicide medium throughout the entire growth period, i.e., both acid and alkaline phases. The fungicide inhibited the occurrence of turbid zones, which appeared in control colonies after seven days of growth, increasing their size with the age of culture. On the medium with Mospilan^®^, *C. albicans* colonies intensively increased their biomass from day three, and the initial alkaline phase was not interrupted by the acid phase and continued throughout the culture. Mospilan^®^ additionally caused differences in the appearance of turbid paths observed after 12 days of culture. As a result of adding Calypso^®^ insecticide to the medium, the production phase of ammonia was observed, which continued throughout the entire cultivation period. No acid phase was observed. The colonies had a smooth phenotype throughout their growth, and their biomass increased with time relative to control. The occurrence of turbidity phases around the colonies was also limited by insecticides, and a small amount around them was visible in 12-day-old colonies.

The Tango^®^ fungicide completely inhibited the growth of *C. pulcherrima* (fungicidal effect) (Figure 3B). On media with Mospilan^®^ and Calypso^®^, the colonies maintained the acid phase throughout the entire cultivation period (no alkaline phase was observed). Colony biomass and phenotype were characteristic of the acid phase, while cell proliferation after three days was much higher in Mospilan^®^ and Calypso^®^ medium compared to control.

As on *C. pulcherrima*, the Tango^®^ fungicide also showed a fungicidal effect on *C. glabrata* (Figure 3C). After exposure to Mospilan^®^ *C. glabrata* resembled the pattern of colony differentiation under control conditions; the alkaline phase appeared earlier (day 12) compared to control (day 15). In addition, an intensive increase in colony biomass could be observed from the very beginning of culture. Similarly, the Calypso^®^ insecticide made it resemble the pattern of colony differentiation under control conditions. However, the extension of the acid phase of colony development and shortening of the phase associated with the production of ammonia could be seen. Additionally, no turbid zones were observed between the colonies.

In the case of *C. tropicalis*, the most abnormal metabolic pattern of the colonies was produced by the Tango^®^ fungicide (Figure 3D), where the acid phase was extended to 12 days. However, no first alkaline phase characteristic of the metabolic pattern of control colonies was observed. Fungicide changed the color of colonies to grey, as well as their morphologies. No turbidity zones present in the control colony system were observed. Interestingly, the morphology of the colonies with all tested pesticides on the 50th day of culture had a lichen structure. The presence of Mospilan^®^ accelerated the occurrence of the alkaline phase as well as increased colony biomass. Moreover, no acid phase was observed. The metabolic pattern of colonies on the Calypso^®^ insecticide medium did not differ from that of the control system.

We conclude by selecting subpopulations of fenpropimorph and epoxiconazole resistant cells in *C. albicans* and *C. tropicalis,* Tango^®^ prolonged the acid phase in the development of a nutrient-metabolizing colony in solid medium. In contrast, a faster developmental transition of colonies to the ammonia-producing phase in *C. glabrata* is connected with the reprogramming of the cellular metabolism, enabling an escape from oxidative stress caused by Mospilan^®^ [41,42]. It can be assumed that in the case of the *C. pulcherrima* colony, the absence of an alkaline phase during growth indicates accelerated cell aging.

**Figure 4:**
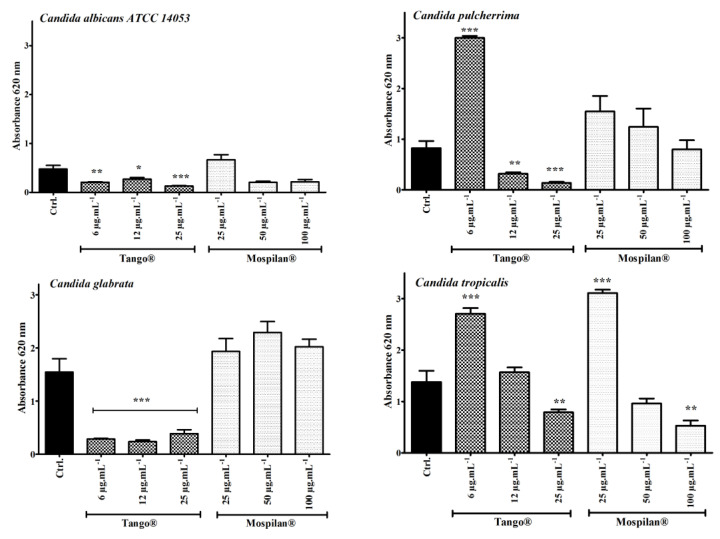
Biofilm formation of *C. albicans ATCC 14053*, *C. pulcherrima*, *C. glabrata*, *C. tropicalis* after Tango^®^ and Mospilan^®^ treatment. Biofilm formation was quantified using crystal violet staining. Bars represent SD, *n* =3, statistical significances after comparison with control *** *p* < 0.001, ** *p* < 0.01, * *p* < 0. Ctrl, control conditions (black); 6, 12, 25 µg·mL^−1^ concentration of Tango^®^, 25, 50, 100 µg·mL^−1^ concentration of Mospilan^®^.

In the next stage of research, each concentration of pesticides was used to determine their effect on *Candida* biofilm formation and development (Figure 4).

Biofilm formation by pathogenic *Candida* yeast is considered the main virulence factor, protecting the pathogen against adverse environmental conditions, mechanisms of the host’s immune response, as well as against the targeted action of antifungal agents [43]. *Candida* biofilms are heterogeneous structures existing as three-dimensional populations of yeast, pseudo-hyphae, and hyphae, embedded within a self-produced extracellular matrix [44].

For all analyzed strains, the highest used concentration of Tango^®^(25 µg·mL^−1^) showed the greatest reduction of biofilm biomass, as assessed by means of a cell viability assay. On the other hand, in the case of *C. pulcherrima* (an increase of about 263.4%) and *C. tropicalis* (an increase of about 96.6%), the lowest used Tango^®^ concentration (6 µg·mL^−1^) stimulated the formation of biofilm compared to control. The highest concentration of Mospilan^®^ inhibited the ability to form biofilm in *C. albicans* and *C. tropicalis*, however, the lowest concentration tested (25 µg·mL^−1^) stimulated the formation of particularly visible biofilm in *C. tropicalis* (an increase of about 125.8%). Calypso^®^ caused a slight decrease in biofilm biomass production in all tested strains except *C. albicans* (Figure S1). At lower concentrations (60 and 120 µg·mL^−1^), the biofilm biomass was reduced 56% and 41% respectively, while at the highest used concentration (250 µg·mL^−1^) it was stimulated by 62.8% relative to control.

### 3.2. Bioavailability of Pesticides

**Figure 5:**
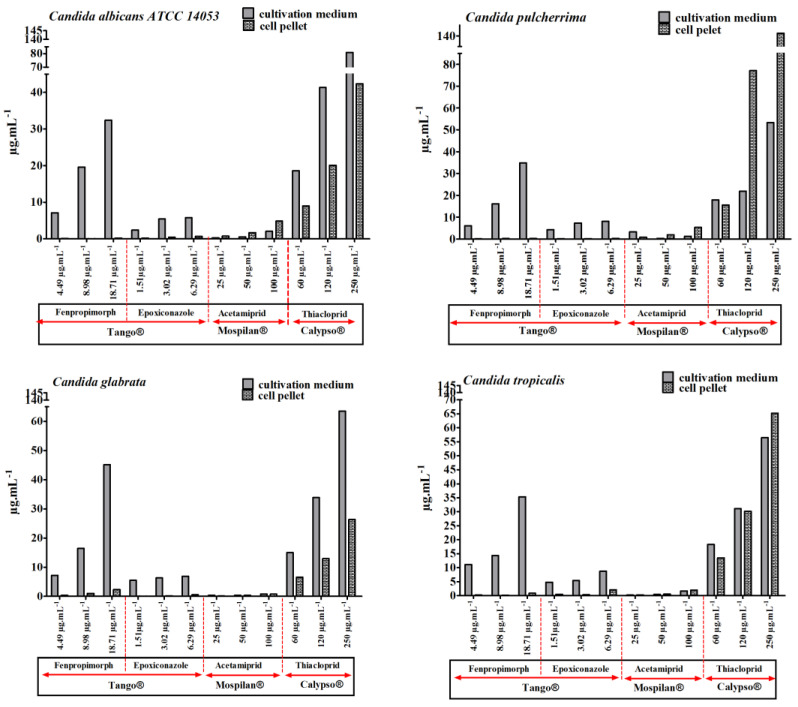
Accumulation of pesticide active agents (fenpropimorph and epoxiconazole in Tango^®^; acetamiprid in Mospilan^®^; thiacloprid in Calypso^®^) in cell pellet and cultivation medium was estimated using gas chromatography with mass spectroscopy (GC-MS).The values are expressed as means, *n* =3.

Additionally, the bioavailability of active agents was estimated (Figure 5). Increased penetration of thiacloprid into *C. pulcherrima* and *C. tropicalis* cells was observed at the highest concentrations of Calypso^®^ insecticide. The concentration of thiacloprid in the cell pellet exceeded the concentration in the supernatant for *C. pulcherrima* by 55.17 µg·mL^−1^at 120 µg·mL^−1^ reaching the value 77.12 µg·mL^−1^ and by 87.95 µg·mL^−1^ at 250 µg·mL^−1^ reaching the value 141.23 µg·mL^−1^. For *C. tropicalis*, the difference was 8.7 µg·mL^−1^ at 250 µg·mL^−1^ reaching the value 65.21 µg·mL^−1^ in the cell pellet. Increased bioavailability of thiacloprid was also observed in *C. albicans* and *C. glabrata*, however, the concentration of the active compound did not exceed those in the supernatant. Similarly, accumulations of acetamiprid in cells of *C. albicans* by 2.82 µg·mL^−1^ (reaching the value 4.87 µg·mL^−1^) and *C. pulcherrima* by 4.01 µg·mL^−1^ (reaching the value 5.29 µg·mL^−1^) were also clearly observed at the highest concentration of 100 µg·mL^−1^ Mospilan^®^ compared to the supernatant. The lowest bioavailability of active compounds was observed with Tango^®^ fungicide. Slight accumulations in the cell biomass of *C. tropicalis* and *C. albicans* were observed in the case of epoxiconazole and of fenpropimorph in *C. glabrata*. In our study, the decrease in amounts of Tango^®^ active compounds observed in *Candida* cells confirms the observations of Esquivel et al. [45] indicating that reduced intracellular accumulation of antifungal agents is a mechanism of drug resistance in many species of fungi. Intracellular accumulation of azole drugs in *Magnaporthe oryzae* depended on the nutrient composition as well as the cell phase of the culture. It has been observed that drug accumulation in older, exponential or stationary growing cells is reduced compared to exponentially growing cells. Adaptation of the culture to various growth media can modulate the composition of the cytoplasmic membrane in cells, and consequently affect azole uptake. In another study, glucose-containing media reduced final drug accumulation levels, probably due to the activation of glucose-dependent efflux pumps [45], which would confirm our results for the active substances in Tango^®^. Moreover, in studies conducted by Mansfield et al. [46], fluconazole accumulation was inversely correlated with the expression of ATP-dependent efflux pumps in energized *C. albicans* cells. De-energized cells took up fluconazole by facilitated diffusion, and changes in this process may be a concealed mechanism of resistance to azole drugs. Accumulation of acetamiprid and thiacloprid in the tested *Candida* species may indicate the involvement of these compounds in metabolism. It has been shown that *Rhodotorula mucilaginosa IM-2* was able to degrade acetamiprid and thiacloprid by hydrolysis of acetamiprid to the intermediate metabolite IM 1-3, and to hydrolyze thiacloprid to form the corresponding amide derivative [47]. Thiacloprid amide still showed insecticidal activity, which may consequently promote *Candida* intercellular variability.

### 3.3. Cytotoxicity and Changes in the Cell Cycle

**Figure 6:**
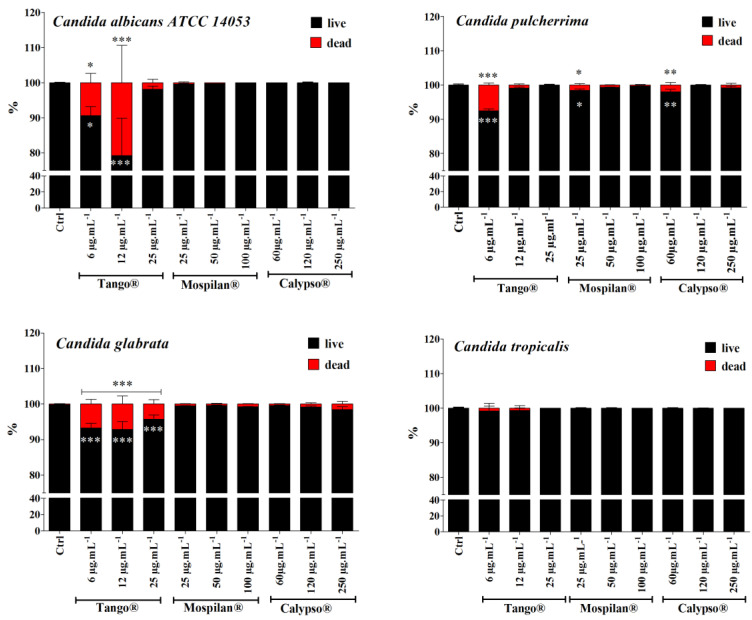
Changes in cell viability of *C. albicans* ATCC 14053, *C. pulcherrima*, *C. glabrata*, *C. tropicalis* cells after pesticide treatment. The percentages of live and dead cells are shown. Bars indicate SD, *n* = 200. *** *p* < 0.001, ** *p* < 0.01, * *p* < 0.05 compared to the viability of control cells (ANOVA + Dunnett’s a posteriori test).

**Figure 7:**
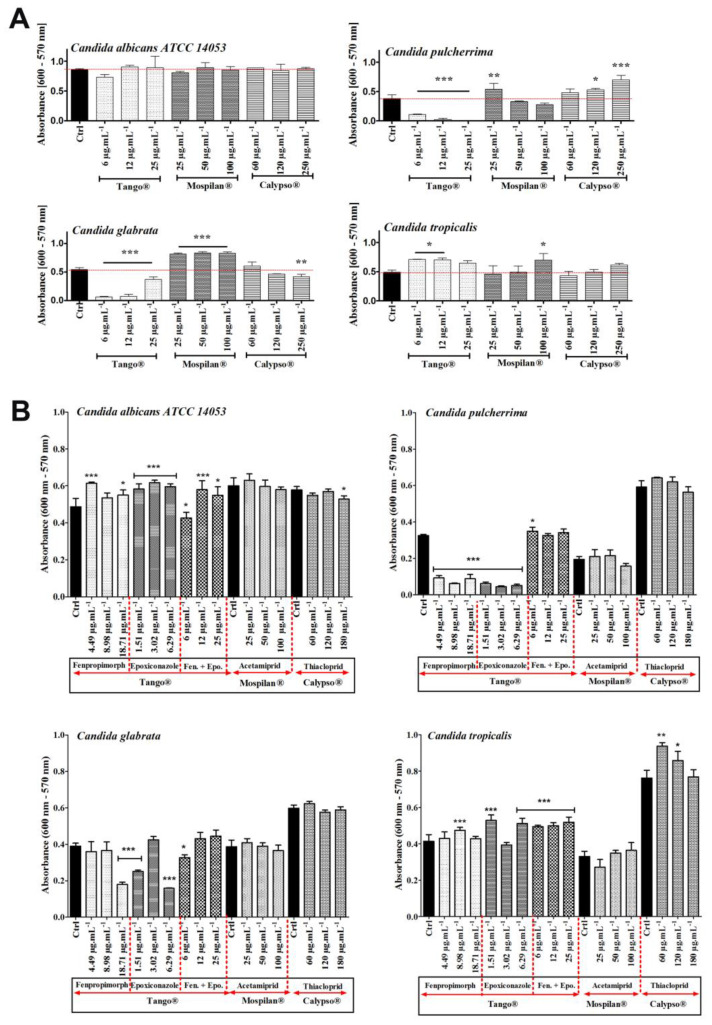
Changes in metabolic activities (**A**, **B**) of *C. albicans ATCC 14053*, *C. pulcherrima*, *C. glabrata, C. tropicalis* cells after pesticide treatment. (**A**) Mean values of metabolic activity of *Candida* spp. cells were estimated with the Alamar Blue (resazurin) assay at different concentrations of pesticide treatment (6, 12, 25 µg·mL^−1^ of Tango^®^; 25, 50, 100 µg·mL^−1^ of Mospilan^®^; 60, 120, 250 µg·mL^−1^ of Calypso^®^), Ctrl, control conditions (black). Bars indicate SD with standard deviations, *n* = 3, *** *p* < 0.00, ** *p* < 0.01, * *p* < 0.05 compared to control (ANOVA + Dunnett’s a posteriori test). (**B**) Mean values of metabolic activity of *Candida* spp. cells were estimated with the Alamar Blue (resazurin) assay after exposure to different concentrations of the pesticides’ active agents (Tango^®^-fenpropimorph (4.49, 8.98, 18.71 µg·mL^−1^), epoxiconazole (1.51, 3.02, 6.29 µg·mL^−1^) and mixture of both; Mospilan^®^-acetamiprid; Calypso^®^-thiacloprid) Ctrl, control conditions (black). Bars indicate SD with SD, *n* = 3, *** *p* < 0.001, ** *p* < 0.01, * *p* < 0.05 compared to control (ANOVA + Dunnett’s a posteriori test).

The tested insecticides showed the different bioavailability (Figure 5); however, changes in the growth of *Candida* strains were observed (Figure 1, Figure 2 and Figure 4). An interest was if these chemicals could change the viability (Figure 6) of the strains and especially their metabolic activity (Figure 7).

In the next stage of research, we decided to determine the effects of the pesticides on *Candida* spp. cell survival rate as well as on the cell cycle. According to the prediction, the Tango^®^ fungicide concentrations used decreased the cell survival rate for *C. albicans*, *C. pulcherrima* and *C. glabrata* (Figure 6). A slight increase in the fraction of dead cells was observed in *C. tropicalis*. The largest fraction of *C. pulcherrima* dead cells was observed after applying Mospilan^®^ at a concentration of 25 µg·mL^−1^. In the case of Calypso^®^ insecticide, the highest mortality rate was observed among *C. pulcherrima* (60 µg·mL^−1^; *p* ˂ 0.01) and *C. glabrata* (250 µg·mL^−1^).

The cytotoxic effects of selected concentrations of the pesticides, as well as their active agents, were checked using the resazurin reduction test (RRT) (Figure 7).It has been shown that Tango^®^ fungicide inhibited the metabolic activity of *C. albicans* (at the lowest concentration of 6 µg·mL^−1^); *C. pulcherrima* and *C. glabrata* cells (in all tested concentrations). *C. pulcherrima* and *C. glabrata* turned out to be the most sensitive species to Tango^®^. No Tango^®^ cytotoxic effect was observed in *C. tropicalis*. On the other hand, an increase in metabolic activity was observed at the lowest Mospilan^®^ concentration, as well as at any Calypso^®^ concentration used during *C. pulcherrima* treatment. A stimulating effect was also observed in *C. glabrata* at all Mospilan^®^ used concentrations, as well as in *C. tropicalis* at 100 µg·mL^−1^. A higher sensitivity to the insecticide concentrations of Mospilan^®^ was seen in *C. pulcherrima* (at 100 µg·mL^−1^) and in *C. glabrata* cells after treated Calypso at 250 µg·mL^−1^. Similarly, a higher sensitivity to Calypso^®^ has been shown in *Cryptococcus laurentii* [48]. Ambiguity of the results in our test for the metabolic activity of *C. albicans* and *C. tropicalis* cells treated with Tango^®^ may suggest the presence of subpopulations of resistant cells with normal metabolic activity and a subpopulation of sensitive cells with low levels of mitochondrial enzymes. An increased sensitivity to fungicide is characteristic of *C. pulcherrima,* where sensitivity to every active compound has been demonstrated. Antagonistic effects have been found when compounds are mixed. Esquivel et al. [45] indicated that antagonism between active compounds may be the result of competition for the import of active compounds into host cells. In the case of insecticides, reduced cytotoxicity is observed in all tested species, when treated either with commercial preparations and/or with the active compounds included in them.

Cytotoxic analysis of the active pesticide compounds showed the dominant activity of fenpropimorph and epoxiconazole contained in Tango^®^ fungicide (Figure 7B). Cytotoxic activity for *C. albicans* cells was visible in the case of the mixture of these compounds corresponding to a concentration of 6 µg·mL^−1^. A strong cytotoxic effect of each of these compounds was observed in *C. pulcherrima* and *C. glabrata* cultures. In the case of their penetration into the human or animal body, they can lead to microbiological flora disorders. A reduction in the number and diversity of bacterial intestinal flora in rats was demonstrated after administration of the triazole fungicide penconazole, which may subsequently translate into the increased ability of a portion of the fungal pathogen cell population to colonize various niches within the mammalian host [49].

*C. pulcherrima* also had reduced cellular metabolic activity due to acetamiprid (100 µg·mL^−1^). *C. tropicalis* was the least sensitive strain, showing a slight decrease in metabolic activity after the application of epoxiconazole and acetamiprid.

**Figure 8:**
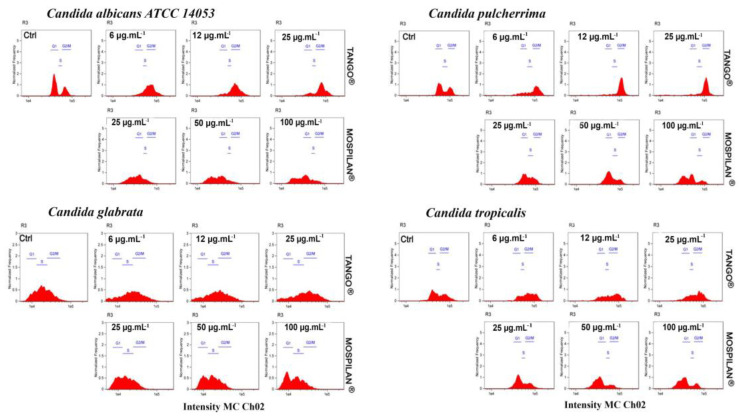
Changes in cell-cycle analysis of *C. albicans ATCC 14053, C. pulcherrima, C. glabrata, C. tropicalis* cells after pesticide treatment. Representative histogram data of cell-cycle analysis of *Candida* spp. cells after pesticide exposure are shown. Histograms represent nuclear DNA content (X axis) relative to normalized cell number (Y axis). Analysis was performed using Amnis^®^FlowSight^®^ flow cytometer and IDEAS software version 6.2.187.0 (Merck Millipore, Warsaw, Poland).please also explain different color means in the picture: red.

In addition, cell-cycle phase analysis of *C. albicans* (Figure 8) after a 24-h incubation of cells with Tango^®^ pesticide revealed a significant decrease in the number of cells in the G1 phase, with a slight increase in the G2/M phase relative to control. In the case of Mospilan^®^ and Calypso^®^ pesticides, a decrease in cell subpopulation was observed in each of the cycle phases, most pronounced in the G2/M phase. Cell-cycle phase analysis of *C. pulcherrima* after Tango^®^ treatment showed an increase in the number of cells in the G2/M phase, with a simultaneous decrease in the G1 phase in a concentration-dependent manner. In the case of Mospilan^®^ at a concentration 100 µg·mL^−1^, the number of apoptotic cells increased with a simultaneous decrease in the number of cells in individual phases. In *Candida glabrata,* the Tango^®^ fungicide reduced cell populations in each of the cycle phases, most pronounced in G1 and S phases. An emergence of apoptotic cell populations was also observed. After Mospilan^®^ and Calypso^®^ treatment, an increase in the number of cells in the G1 phase was observed at the highest concentrations of the compounds used (100 µg·mL^−1^ Mospilan^®^ and 250 µg·mL^−1^ Calypso^®^) (Figure 8 and Figure S2). Cell-cycle analysis of *C. tropicalis* showed an increase in the G2/M cell population after Tango^®^ exposure at each concentration and a decline in the G1 phase. Mospilan^®^ reduced the number of cells in M and G2/M phases, most clearly at concentrations 50 µg·mL^−1^ and 100 µg·mL^−1^. Calypso^®^ did not inhibit any of the cell-cycle phases (Figure S2).

### 3.4. Oxidative Stress and DNA Damage

**Figure 9:**
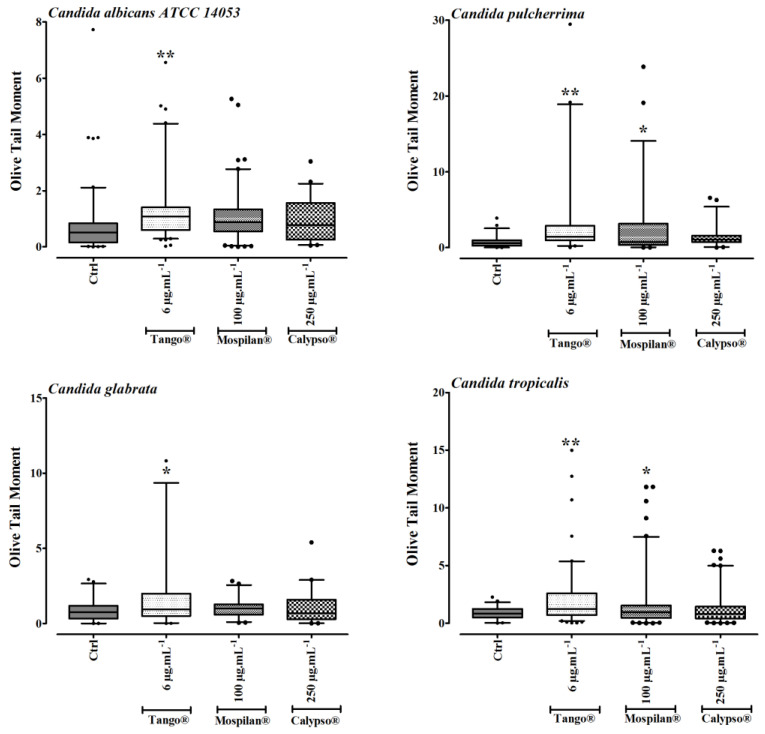
Induction of DNA damage assessed with the alkaline comet assay (olive tail moment) in *C. albicans ATCC 14053*, *C. pulcherrima*, *C. glabrata, C. tropicalis* cells after Tango^®^(6 µg·mL^−1^), Mospilan^®^ (100 µg·mL^−1^) and Calypso^®^(250 µg·mL^−1^) treatment. Bars indicate SD, *n* = 200, box and whisker plots are shown, ** *p* < 0.01, * *p* < 0.05 compared to control conditions (ANOVA and Dunnett’s a posteriori test). Ctrl—control conditions. Notes: Center line represents median. Lower and upper limits represent 5th and 95th percentiles, respectively. Observed values outside whiskers shown as dots.

For DNA damage detection, the alkaline comet assay was used (Figure 9, Figure S3). In all *Candida* species Tango^®^ was able to induce significant levels of DNA breaks (Figure 9) at a concentration of 6 µg·mL^−1^ (*p* < 0.01, *p* < 0.05). Mospilan^®^ caused a significant increase in DNA breaks at a concentration of 100 µg·mL^−1^ (*p* < 0.05), observed in *C. pulcherrima* and *C. tropicalis*. An increase in DNA damage was also observed after Calypso^®^ treatment in all tested species, the highest level in *C. pulcherrima*.

**Figure 10:**
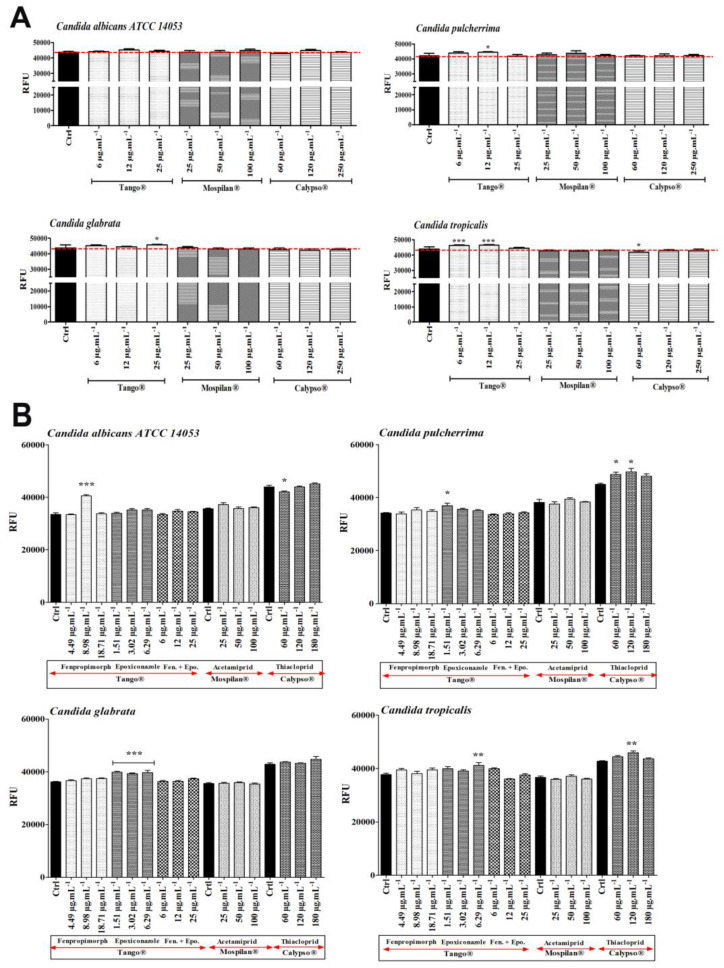
(**A**) Assessment of oxidative stress damage in *Candida* spp. cells after pesticides treatment (6, 12, 25 µg·mL^−1^ Tango^®^; 25, 50, 100 µg·mL^−1^ Mospilan^®^; 60, 120, 250 µg·mL^−1^ Calypso^®^). (**B**) Assessment of oxidative stress damage in *Candida* spp. cells after treatment with different concentrations of pesticides’ active agents (Tango^®^-fenpropimorph (4.49, 8.98, 18.71 µg·mL^−1^), epoxiconazole (1.51, 3.02, 6.29 µg·mL^−1^) and a mixture of both; Mospilan^®^—acetamiprid (25, 50, 100 µg·mL^−1^); Calypso^®^—thiacloprid (60, 120, 180 µg·mL^−1^). MitoTracker^®^ Red CM—H2Xros was used in both to evaluate mitochondrial superoxide levels. Bars indicate SD with standard deviations, *n* = 3, *** *p* < 0.001, ** *p* < 0.01, * *p* < 0.05 compared to control (ANOVA + Dunnett’s a posteriori test). Ctrl, control conditions (black).

In connection with the demonstrated increase in the levels of DNA damage in the next stage of research, it was checked whether their cause is the accumulation of reactive oxygen species (ROS) during pesticidal stress. Oxidative stress damage was analyzed by means of mitochondrial superoxide levels in the *Candida* cells treated with commercial pesticides (Figure 10A) as well as their active agents (Figure 10B). Significantly elevated levels were observed in *C. tropicalis* cells after Tango^®^ treatment (Figure 10A; 6 and 12 µg·mL^−1^; *p* < 0.001). A slight increase in peroxide levels with no statistical significance was observed in *C. albicans* after exposure to Tango^®^ (12 µg·mL^−1^), Mospilan^®^ (100 µg·mL^−1^) and Calypso^®^ (120 µg·mL^−1^). An increase in peroxide levels was also observed in *C. pulcherrima* cells after Tango^®^ (6 and 12 µg·mL^−1^; *p* < 0.05), and Mospilan^®^ (25 and 50 µg·mL^−1^) exposure as well as in *C. glabrata* cells after Tango^®^ (6 and 25 µg·mL^−1^; *p* <0.05) exposure.

Second, the effects of active pesticide compounds were investigated with regard to oxidative stress damage (Figure 10B). Analysis of *C. albicans* cells showed a significant increase in fenpropimorph-induced peroxides at 8.98 µg·mL^−1^ (*p* < 0.001) and a slight increase after epoxiconazole treatment at 3.02 and 6.29 µg·mL^−1^. In addition, a slight increase was observed at 25 µg·mL^−1^ acetamiprid, without statistical significance. In contrast, in the case of *C. pulcherrima* cells, the increase in peroxide level was caused by epoxiconazole treatment at a concentration of 1.51 µg·mL^−1^ as well as by thiacloprid at all concentrations tested, with the maximum at concentration 120 µg·mL^−1^ (*p* < 0.05). In *C. glabrata* a significant increase (*p* < 0.001) in peroxide levels at all epoxiconazole concentrations was observed. A slight increase in peroxide levels in the analyzed *C. tropicalis* cells was observed at selected concentrations of fenpropimorph and epoxiconazole, and for mixtures of these compounds at a concentration corresponding to 6 µg·mL^−1^ of commercial Tango^®^ pesticide. A significant increase in peroxide levels was caused by thiacloprid at a concentration of 120 µg·mL^−1^ (*p* < 0.01).

### 3.5. Metabolic Activity Changes

**Figure 11:**
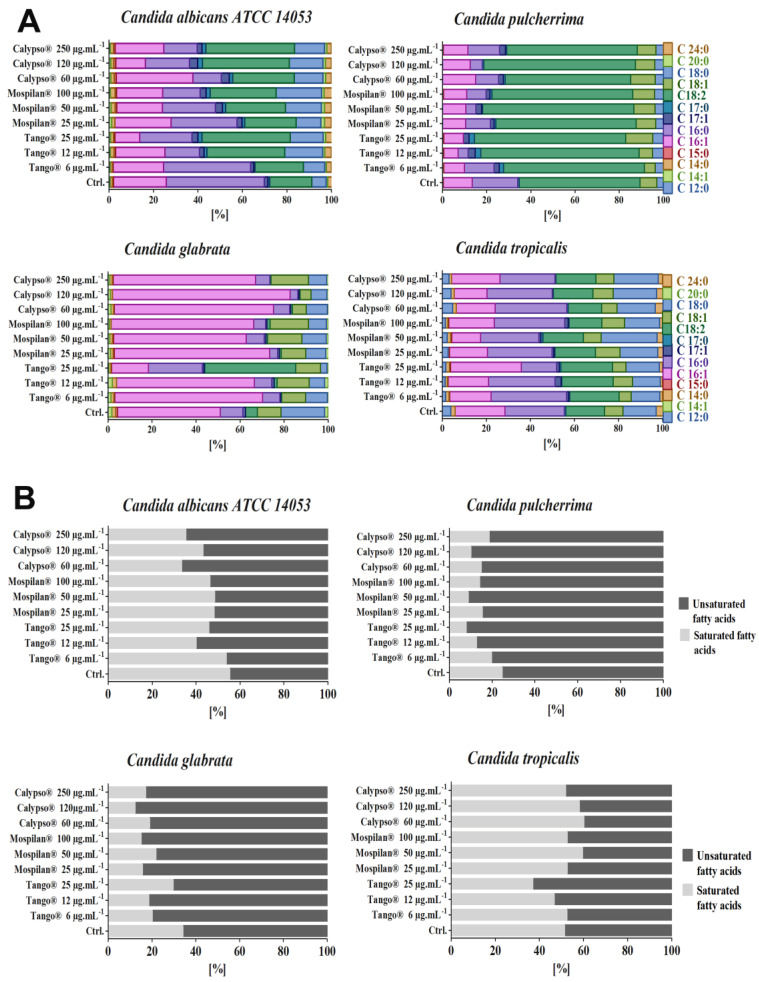
(**A**) Fatty acid profiles and (**B**) unsaturated/saturated indices in *Candida* spp. cells after pesticide treatment. (**A**) Fatty acid distribution after pesticide treatment was estimated using gas chromatography with a mass detector in full scan mode. (**B**) The unsaturated index was calculated as the sum of FA weights multiplied by the number of unsaturated bonds for each FA in the mixture. The saturated index was calculated analogously. Percentage contents of saturated and unsaturated FAs are shown. Ctrl, control conditions; 6, 12, 25 µg·mL^−1^ concentration of Tango^®^, 25, 50, 100 µg·mL^−1^ concentration of Mospilan^®^, 60, 120, 250 µg·mL^−1^ concentration of Calypso^®.^

First, the compositions of individual fatty acids in the studied *Candida* species during pesticidal stress were analyzed (Figure 11A,B). In *C. albicans*, pesticide treatment significantly increased polyunsaturated fatty acid (PUFA) content, whereas it decreased monounsaturated fatty acid (MUFA) and total saturated fatty acid (SFA) contents. These changes were caused by a drop in the amount of palmitoleic (C16:1) and especially visible palmitic (C16:0) acids and a simultaneous increase in the levels of linoleic (C18:2) and stearic (C18:0) acids. In addition, comparison of the FAs of *C. pulcherrima* showed that the amount of C18:2 was increased in all pesticide concentrations applied. The C16:0 percentage increase was particularly evident in the *C. tropicalis* cell population treated with the lowest concentrations of pesticides. In contrast to the others, *C. glabrata* increased the percentage level of palmitoleic (C16:1) acid. The exception was the highest used concentration of Tango^®^ (25 µg·mL^−1^), where a 30% decrease compared to control was observed, while the palmitic acid level (16:0) increased by 14.3% (Figure 11A). Other concentrations of all pesticides used reduced the level of palmitic acid (C16:0). The unsaturation index of FAs in *C. glabrata* cells increased by 28%, 31.3% and 9.1% (6, 12, 25 µg·mL^−1^ Tango^®^ respectively), 37.1%, 24.7% and 38.3% (25, 50, 100 µg·mL^−1^ Mospilan^®^ respectively), and 30.4%, 43.7% and 34% (60, 120, 250 µg·mL^−1^ Calypso^®^ respectively) compared with control. In addition, exposure of *C. pulcherrima* cells to pesticidal stress resulted in an increase in unsaturated FAs compared with control. A similar tendency was also observed to a lesser extent in *C. albicans* cells. An increase in the saturation index of C. *tropicalis* cells was observed with Mospilan^®^ and Calypso^®^ at all concentrations used, in contrast to Tango^®^ concentrations, where the unsaturation index of cells increased (Figure 11B).

**Figure 12:**
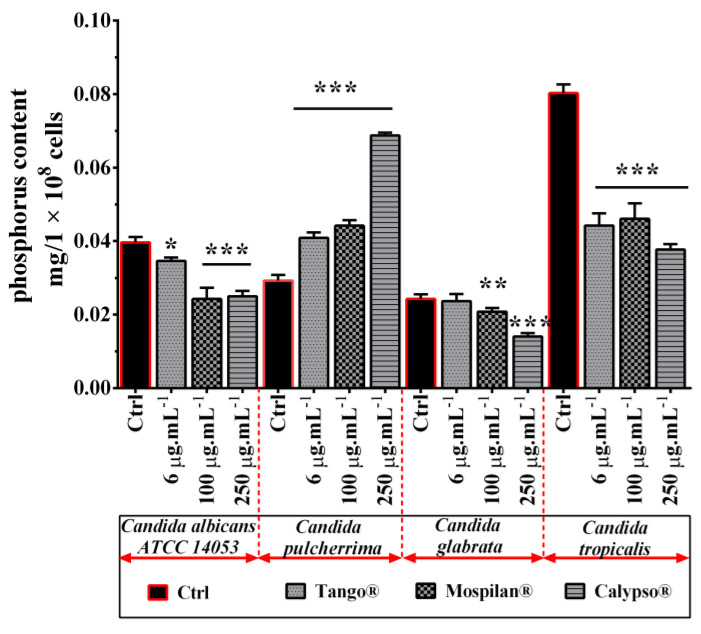
Phospholipid determination based on phosphorus in *Candida* spp. cells after pesticide treatment. The phospholipid content was measured spectrophotometrically in 1 × 10^8^ cells of each species using a phosphorus assay. Bars indicate SD, *n* = 3, *** *p* < 0.001, ** *p* < 0.01, * *p* < 0.05 compared to control (ANOVA and Dunnett’s a posteriori test).

Secondly, the treatment of all *Candida* species cells with selected pesticide concentrations produced changes in their phospholipid profiles (Figure 12). After exposure to 6 µg·mL^−1^ Tango^®^ there was a 36% increase in phospholipid phosphorus content in *C. pulcherrima* cells, but a 13% decrease in *C. albicans* and a 43% decrease in *C. tropicalis* (*p* < 0.001). Exposure to 100 µg·mL^−1^ Mospilan^®^ led to a statistical decrease in phospholipid phosphorus content in *C. albicans*, *C. glabrata* and *C. tropicalis* cells by 30%, 12.1% and 40.2% respectively, but with a simultaneous increase of 51.4% in *C. pulcherrima* cells. A similar situation was observed after treatment with 250 µg·mL^−1^ Calypso^®^, where an increase in phospholipid phosphorus content by 127.5% was observed in *C. pulcherrima* and a decrease by 32.6% in *C. albicans*, 37% in *C. glabrata*, and 52.6% in *C. tropicalis* (Figure 12).

Furthermore, pesticide treatment did not induce changes in glycogen accumulation by *C. albicans* cells (Figure S4). A slight increase in the number of glycogen accumulation cells was observed in *C. tropicalis* at concentrations of 12 and 25 µg·mL^−1^ Tango^®^ and 120 and 250 µg·mL^−1^ Calypso^®^. *C. pulcherrima* and *C. glabrata* cells did not accumulate glycogen (Figure S4).

## 4. Discussion

Given that the source of candidiasis is an endogenous infection, pesticides entering the body are underestimated factors that may play an important role in developing cellular diversity and affect *Candida*–host interactions during colonization. The heterogeneity of cells can have different properties and lead to multiple benefits for the population [50]. Inhomogeneity in the population includes genetic and metabolic diversity that translates into different cellular phenotypes observed in studies on morphology, survival, biofilm-forming ability and cell cycle changes. At the beginning of this study, we analyzed the impact of the pesticides tested on the morphological profile of selected species of the genus *Candida*. It is recognized that in the population of fungi both yeast and pseudohyphae are similar in morphology, and in the case of only three, phylogenetically closely-related species, i.e., *C. tropicalis*, *C. dubliniensis* and *C. albicans*, may also appear in the form of hyphae [51,52]. *C. glabrata* is the only pathogenic species that does not produce filamentous forms, existing exclusively as blastoconidia [53]. The morphological transition of yeast into filamentous form in *C. tropicalis* was observed in the presence of all tested concentrations of pesticides and can be equated with the invasiveness of this species, referred to as virulence [43,51]. Despite the morphological plasticity demonstrated by the researchers in *C. albicans*, the pesticides used in this study did not stimulate morphological changes. It proved that fluconazole-resistant strains suppressed induction of hyphal formation due to the influence of the antibiotic in the amount of MIC, which did not inhibit cell culture growth, although the change in morphological forms was limited [54,55]. It has also been demonstrated that for some fungal pathogens including *C. glabrata*, there is no morphological transition of the pathomechanism from commensal to pathogen. Our analysis of cell growth kinetics after Tango^®^ and Mospilan^®^ treatment showed a longer adaptation phase in the growth curve for the populations of *C. albicans*, *C. pulcherrima* and *C. glabrata*. Other studies have not shown any inhibitory effect on cell division by Calypso^®^. Moreover, a stimulatory effect on yeast growth was observed after treatment of yeast cultures with iprodione pesticide, where it was shown that the ingredients of the fungicidal preparation may become an additional source of energy for bacteria and yeasts by increasing the biomass of the culture [56].

Additionally, the active substances in Tango^®^, epoxiconazole and fenpropimorph, increased the dead cell fraction in all tested *Candida* strains, except *C. tropicalis*. In case of their penetration into the human or animal body, they can lead to microbiological flora disorders. A reduction in the number and diversity of bacterial intestinal flora in rats was demonstrated after the administration of the triazole fungicide penconazole, which may subsequently translate into increased ability of a portion of the fungal pathogen cell population to colonize various niches within the mammalian host [49].

It seems that determining the impact of the tested pesticides on biofilm formation may be a measure of the ability of the tested yeasts to adapt to stress conditions. Biofilm formation is a complex process involving many types of cells and stages [57]. An increase in biofilmation at the lowest fungicide concentrations may affect the transcription of resistance genes in the clonal cell population. Heterogeneous resistance, or selection for a resistant subpopulation of cells, has been well documented in *Candida albicans* [58] or *Cryptococcus neoformans* [59]. It is suggested that the increased metabolic activity occurring in the early development of biofilm in *Candida* species contributes to their greater resistance to antifungal drugs. Another reason is the presence of persister cells that do not divide but maintain high tolerance to antimicrobial drugs. The presence of these cells allows antimicrobial drugs to bind to their specific target, preventing the drug from inhibiting the function of the target molecule [57].

Observed cell heterogeneity may also be a result of disorders in the cell cycle of the analyzed strains after treatment with selected concentrations of pesticides [60]. In addition, the heterogeneity of cells to oxidative stress responses and increased levels of ROS can enhance the potential of interacting with DNA and cause genotoxicity leading to recombination and diverse mutations [61,62]. Our analysis showed ROS increase in all *Candida* species, especially after the treatment of cells with Tango^®^, both in commercial pesticide form and also after the application of the active compounds included in it. We also observed an increase in total DNA damage in the cells of the analyzed species (*p* < 0.01, *p* < 0.05), most clearly visible with Tango^®^. It has been demonstrated that antifungal agents such as amphotericin B and azoles trigger common cell-death pathways causing oxidative damage in fungi such as *Candida albicans*, *Saccharomyces cerevisiae* or *Cryptococcus gattii* [63]. Supporting our results, it was found that Tango^®^ [33] and a thiacloprid-based insecticide induced oxidative stress in bovine lymphocytes in other studies [64]. It appears that modulation of ergosterol synthesis caused by the twin-component fungicide (fenpropimorph and epoxiconazole) in all studied *Candida* strains could play a key role in the adaptation of cells during oxidative stress [65]. The oxidative and nitrosative stress induced by azoles (miconazole, fluconazole) in many clinical *C. albicans* isolates was identified as a factor determining their sensitivity to miconazole [66]. Interestingly, it has been indicated that noise in the expression of genes involved in DNA replication, repair and recombination processes can directly cause heterogeneity among cells in terms of mutation rate and/or recombination, which would also have consequences for the occurrence of cells pre-adapted to the given environment [67]. In addition, changes in the metabolic profile of the colony during aging can lead to changes in the structure of populations which differ in their resistance to stress, cell metabolism, and respiration and ROS production especially. The aging colony process in yeast is of particular importance in relation to pathogenic fungi, because the accumulation of old cell subpopulations can lead to increased resistance to attack by host immune cells or the action of drugs [68].

Further analysis of the fatty acid profiles of the *Candida* species studied in response to the applied pesticidal stress revealed cells’ phospholipid profile remodeling estimated on phospholipid phosphorus content, as well as changes in the percentage of unsaturated to saturated FA, increasing the likelihood of differences between the cells. It was found that the overall response to pesticidal stress in the analyzed *Candida* species (to a lesser extent in *C. tropicalis*) is the accumulation of subpopulations of cells with increased fatty acid unsaturation rates. Our findings during inducted pesticidal stress showed differences in the amounts of saturated fatty acids, i.e., palmitic acid (C16:0) and stearic acid (C18:0), and of unsaturated fatty acids, i.e., palmitoleic acid (C16:1) and linoleic acid (C18:2). Considering the similarity of Tango^®^ functioning to the mechanism of azoles group action, it appears that the pesticides used may similarly affect phospholipid and fatty acid profiles, which has been confirmed in another study [69]. Similarly, a higher percentage of unsaturated compared to saturated fatty acids observed not only in the case of the fungicide used but also insecticides, may increase the fluidity of the cytoplasmic membrane *Candida* cells. Other studies also indicate that resistance to miconazole was associated with a decrease in total lipids, phospholipids and sterol content [70].

In fluconazole-resistant *C. albicans* strains, higher efflux pump activity was observed as a result of increased membrane fluidity and reduced levels of ergosterol. It was shown that Cdr1p and Cdr2p represent two major drug extrusion pumps in *C. albicans*, effluxing not only azoles and their derivatives but also a wide variety of structurally unrelated compounds. Contrary to our studies, no significant alteration was observed in the phospholipid and fatty acid composition of the investigated *C. albicans* strains [71,72]. It can be assumed that pesticides can also be removed by the mentioned major drug extrusion pumps, but this requires further research.

Fungal pathogens such as *C. albicans* are characterized by extensive metabolic plasticity, which allows them to adapt to the nutritional conditions of the various host habitats [73]. The effect of pesticides on glycogen accumulation in all *Candida* species did not show significant differences from control. *C. pulcherrima* and *C. glabrata* cells showed the lowest glycogen storage capacity. Zeitz et al. [73] indicated that glycogen deficiency does not affect long-term survival, growth, metabolic flexibility or morphology of *C. albicans*. Similar results regarding the *C. glabrata* strain were obtained in our other studies [74].

## 5. Conclusions

In conclusion, we show in our work that, pesticides are still an underestimated source of microorganism variability, which consequently may lead to the development of strains resistant to drugs commonly used to control them. Analyses have shown that insecticides entering from the environment through various routes into organisms can lead to the accumulation of ROS in cells, causing oxidative damage to DNA and consequently can promote *Candida* spp. intercellular variability and may indirectly influence their pathobiology, just like fungicides. Comparison of fatty acid profiles of cells of tested species of the *Candida* genus revealed the remodeling of FAs and unsaturated/saturated index of the FAs during pesticidal stress. The overall response to the fungicide as well as insecticides has been found to be the accumulation of subpopulations of cells with elevated fatty acid unsaturation rates, except for *C. tropicalis.* Particularly interesting is that for the first time the aspect of the tested insecticides on the developmental phases of the colonies of the tested *Candida* spp. during aging, where cells with high density were undergoing metabolic changes may imitate the state of infection in vivo was studied. Therefore, the understanding of the factors modifying the intercellular variation of *Candida* spp. is one of the major challenges of modern science, which may play an important role in the development of new therapeutic strategies.

## Figures and Tables

**Table 1:** List of *Candida* spp. used in experiments.

Strains	Characteristics
*Candida albicans ATCC 14053*	Control strain
*Candida pulcherrima VKM Y-955*	Environmental isolate, strains were kindly provided from the Institute of Cell Biology NASU Lviv, Ukraine
*Candida glabrata*	Urinary isolate, female; Identification (API^®^ Candida Biochemical Test) and DNA sequencing–GenBank Accession Number–LC389261.1
*Candida tropicalis*	Bronchoalveolar lavage isolate, male; Identification (API^®^Candida Biochemical Test) and DNA sequencing–GenBankAccession Number-KX664669.1

## Supplementary Materials

The following are available online at https://www.mdpi.com/2073-4425/11/8/848/s1, Figure S1: Biofilm C; Figure S2: Cell cycle new; Figure S3: Comet foto; Figure S4: GlycogenAuthor Contributions.
