# Supplementary figures and images for: Synthetic Pesticides Used in Agricultural Production Promote Genetic Instability and Metabolic Variability in Candida spp
Source: Genes (Basel). 2020 Jul 24;11(8):848. doi: 10.3390/genes11080848 (PMC7463770; doi:10.3390/genes11080848)

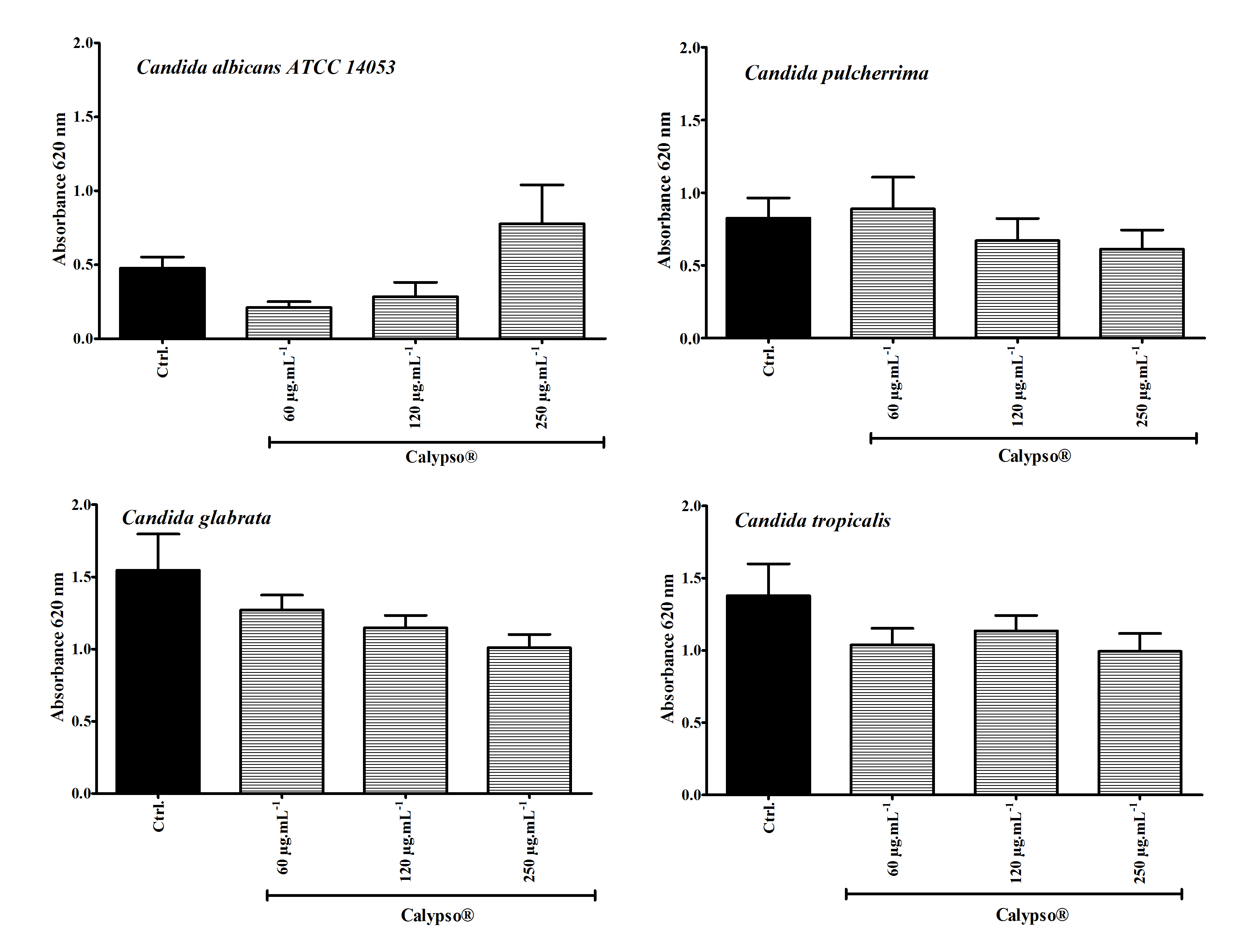

Supplement: Supplementary file 1 [file genes-11-00848-s001.zip › sup/FSuppl. 1 biofilm C.tif]

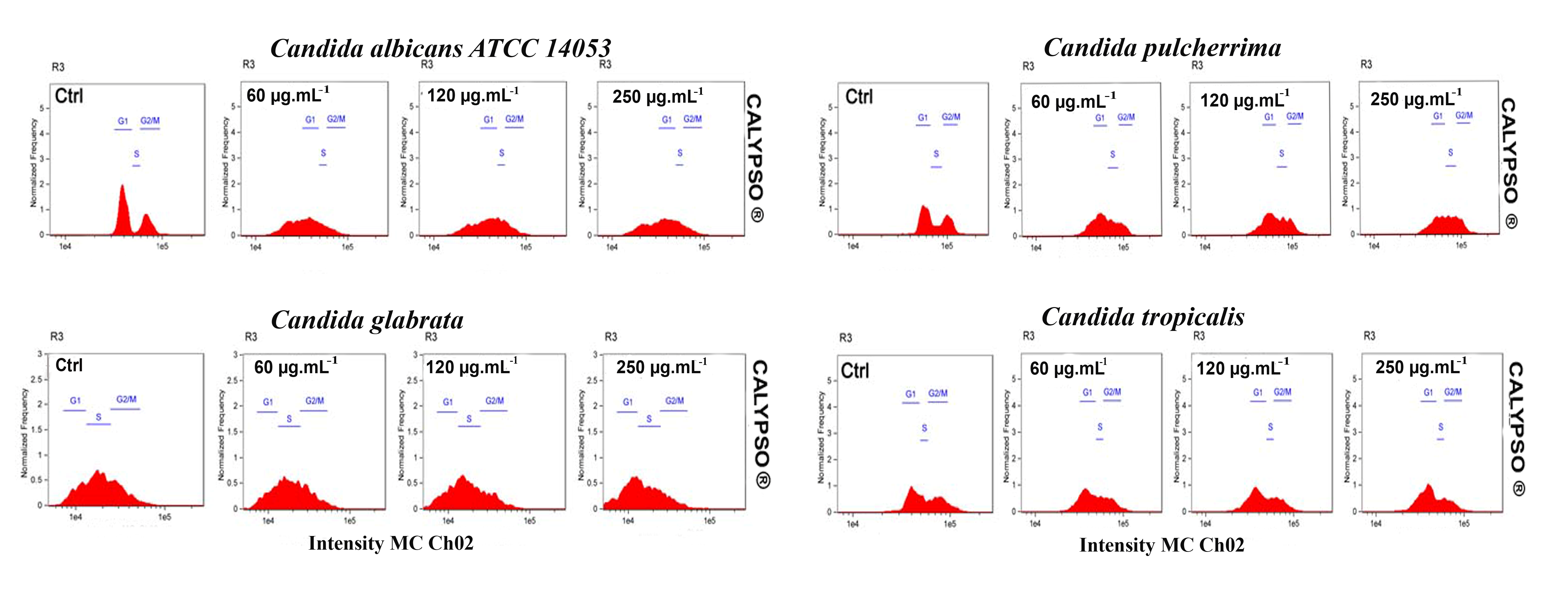

Supplement: Supplementary file 1 [file genes-11-00848-s001.zip › sup/FSuppl. 2 cell cycle new C.tif]

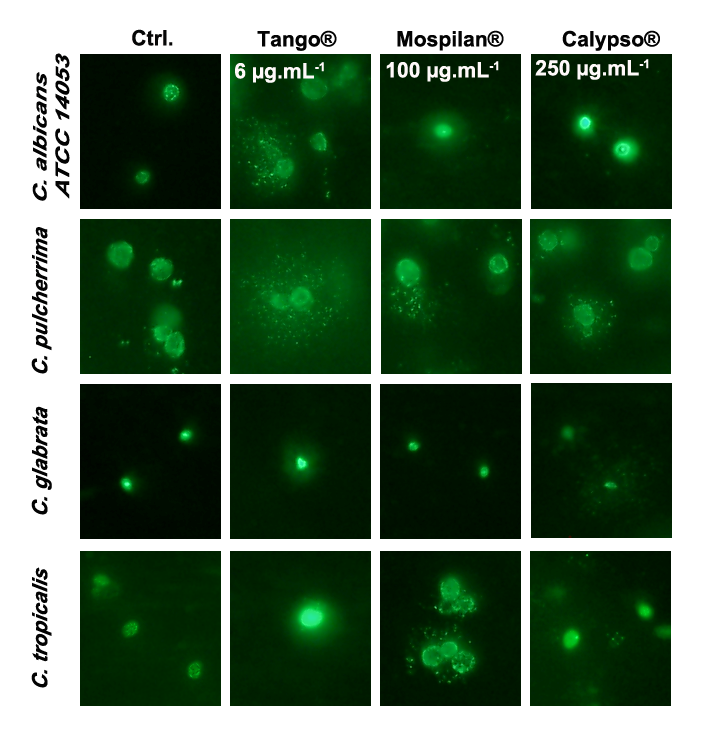

Supplement: Supplementary file 1 [file genes-11-00848-s001.zip › sup/FSuppl. 3 comet foto.tif]

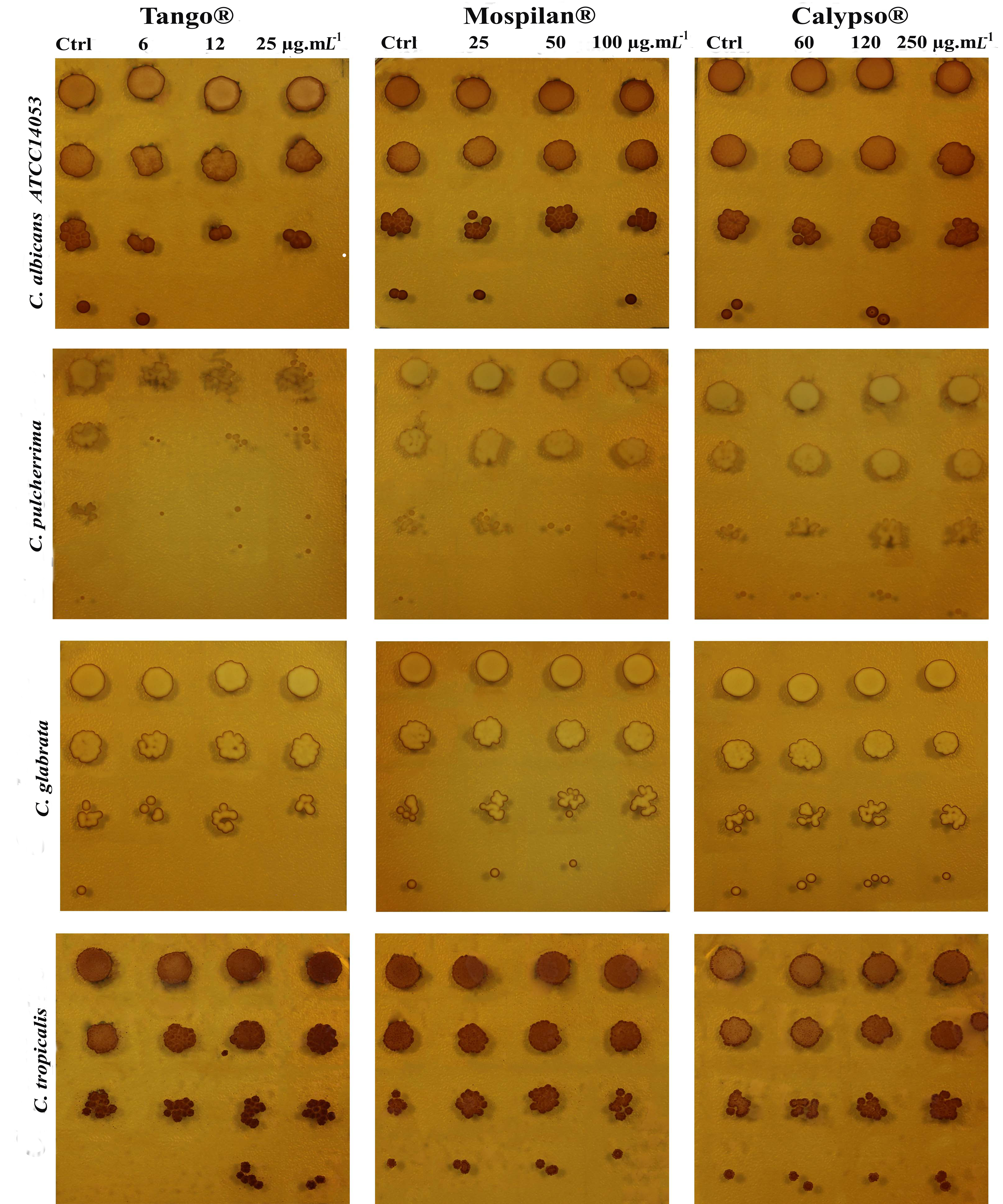

Supplement: Supplementary file 1 [file genes-11-00848-s001.zip › sup/FSuppl. 4 glycogen.tif]
